# Is Niagara River Water Healthy?

**Published:** 1873-09

**Authors:** 


### Is Niagara River Water Healthy?

We have been invited to give opinion upon the hygenic influence of our water supply, a subject which we have thus far carefully avoided. Perhaps in our next number we may be able to give some facts concerning it. Absence of disease directly tracable to its use as cause, and apparent purity in chemical analysis cannot make Hamburgh Canal water good coffee, or satisfy Buffalo people that they can safely drink or advantageously bath in it during at least a portion of the year. Bad and unsatisfactory as it is, we are yet unwilling to misrepresent it, or accuse it of murder or manslaughter unjustly.

